# Revision Surgery After Complications of Silicone Chin Implants

**DOI:** 10.3390/jcm15041326

**Published:** 2026-02-07

**Authors:** Rafał Pokrowiecki

**Affiliations:** 1 Department of Cranio-Maxillofacial Surgery, Oral Surgery and Implantology, Medical University of Warsaw, 02-005 Warsaw, Poland; dr.pokrowiecki@gmail.com; Tel.: +48-691226414; 2 Private Practice, Prive Aesthetic and Facial Feminization Surgery Centre, 02-640 Warsaw, Poland

**Keywords:** angle, augmentation, chin, complications, facial implants, implant fixation, malar, silicone, stabilization

## Abstract

**Background:** Silicone chin implants have been widely used is plastic and esthetic surgery of the face being considered as safe and efficient way for chin augmentation. However, complications such as bone resorption, displacement or ectopic bone formation may occur. **Methods:** The objective of this study was to evaluate complications associated with silicone chin implants and revision surgery protocols. **Results:** Among 98 patients who received silicone chin implants, 24 (11 males, 13 females) exhibited complications. The most commonly diagnosed issues were displacement (*n* = 3), bone resorption (*n* = 9), both conditions (*n* = 3), and patient dissatisfaction (*n* = 7). All patients were qualified for revision surgery, which included silicone implant removal followed by sliding genioplasty (*n* = 7), orthognathic surgery (*n* = 4), custom-made chin implant placement (*n* = 7), and repositioning and fixation (*n* = 1). After revision surgery, no complications occurred. **Conclusions:** Observations from this revision cohort suggest that careful patient selection and consideration of orthognathic or customized implant-based approaches may reduce the risk of dissatisfaction and revision surgery in patients with dentofacial deformities, or those seeking gender confirmation surgeries, compared to stock silicone implants.

## 1. Introduction

Silicone elastomer is a solid, rubber-like polymer composed of polydimethylsiloxane. Silicone facial implants have been established as a versatile tool in contemporary plastic and esthetic facial surgery, as silicone is biocompatible and highly resistant to degradation, with minimal allergic reaction and risk of toxicity [1]. They have been described as an efficient and less-invasive approach to tissue augmentation than osteotomy-based or bone-grafting techniques, with lower complication rates and morbidity [2,3]. The most commonly used implants are for the chin, mandibular angle, and malar region. Chin implants are used in patients with a receding chin due to aging and/or bone deficiency associated with Class II malocclusion; therefore, they are the most frequently used facial implants, providing an immediate “on-table” effect with a relatively low complication rate. They may also be used as an additive after genioplasty when a deepened labiomental sulcus is diagnosed [4]. However, bone resorption, implant displacement, and resulting asymmetry have been reported, highlighting the importance of implant stabilization (Niamtu, 2000, 2009) [5,6]. Unstable implants may cause tissue irritation, encapsulation, bone compression and erosion, and facial asymmetry. The current literature provides limited evidence on alloplastic facial implant stabilization and on fixation protocols (sutures, screws, supra- or subperiosteal implant positioning) (Madorsky & Meltzer, 2025) [7]. Debate continues regarding optimal silicone fixation to prevent these sequelae. This report discusses the clinical manifestations of complications associated with silicone chin implants encountered in the daily practice of plastic and maxillofacial surgeons. It also emphasizes the importance of evaluating malocclusion and considering orthodontic treatment before selecting patients for esthetic surgery.

## 2. Materials and Methods

A retrospective review was conducted on patients who underwent facial reconstruction using silicone facial implants between January 2019 and December 2025.

Eligible patients met all of the following criteria:

(1) Facial reconstruction with the use of silicone implants;
(2) A minimum follow-up period of 6 months;
(3) Availability of complete preoperative and postoperative clinical documentation, including medical records and imaging;
(4) Complications were diagnosed.

Before qualification for the operation, facial CT images were obtained using a Carestream 9600 with the following scanning parameters: 120 kV, 5 mAs, and a 300 × 300 × 300 µm voxel matrix (Atlanta, GA, USA). All procedures were carried out in accordance with the Declaration of Helsinki. The study protocol received approval from the Bioethics Committee of the Medical University of Warsaw (AKBE/21/2025). Written informed consent was obtained from all individuals whose clinical photographs are included in this manuscript.

## 3. Results

**Figure 1:**
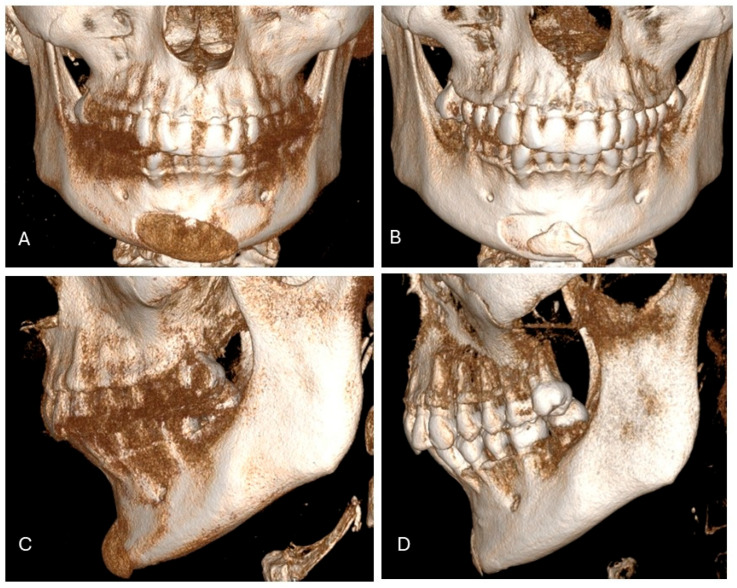
A 42-year-old Caucasian woman presented for consultation because of a mispositioned chin implant placed elsewhere four years earlier (**A**). She complained of asymmetry, palpable implant mobility, tissue irritation, and mild pain. CT showed implant malposition due to lack of fixation (**A**,**C**), with bone resorption and ectopic bone formation (**B**,**D**). The patient was scheduled for implant removal with immediate osteoplasty and placement of a custom-made PEEK chin implant.

**Figure 2:**
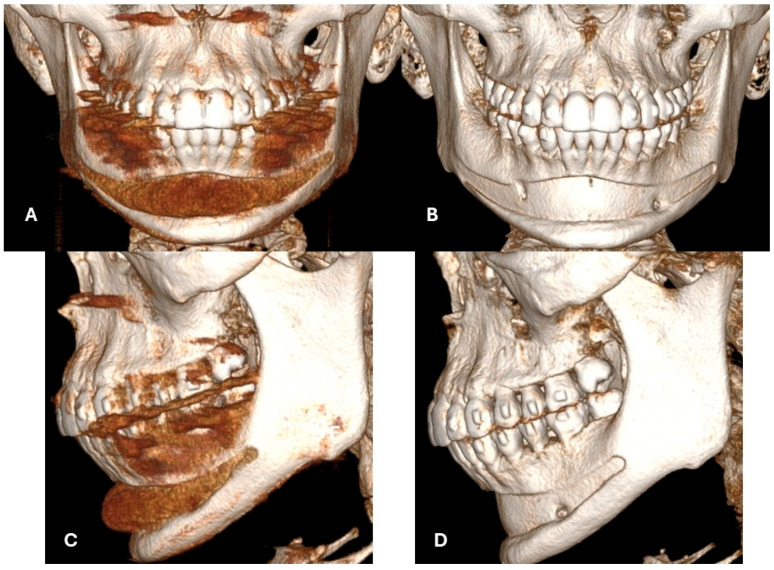
A 21-year-old Caucasian woman who presented for consultation after a failed stock chin-implant placement elsewhere. Clinically, she exhibited Class II malocclusion with vertical overgrowth of the anterior maxilla resulting in a gummy smile, recessed chin, overbite, and retrognathia. She was undergoing orthodontic treatment with removable (camouflage) appliances at the time of consultation. Orthodontic camouflage and implant placement had been attempted to mask the major craniofacial and dental arch relationship anomalies. CT imaging showed the chin implant was positioned inappropriately high relative to the mental protuberance and not on the inferior mandibular border (**A**–**C**). This led to implant collapse into the mandibular groove and inadequate soft-tissue projection (**B**). The implant had been placed passively without stabilization, which caused significant bone resorption along the implant, extending to the apices of the anterior teeth (**B**,**D**).

**Figure 3:**
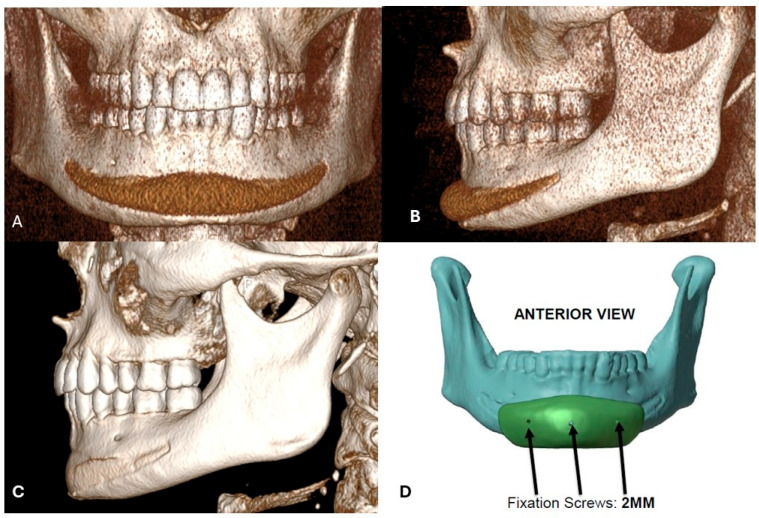
A 30-year-old Caucasian man who presented for consultation after a failed chin implant placed to enhance the lower third’s masculine features. A stock silicone implant had been used and did not provide the expected volumizing effect or masculinization of the mandible (**A**). CT imaging revealed the implant caused bone resorption along its entire length- anterior view and (**B**) lateral view. (**D**). After the digital removal of the silicone implant, reconstructed images confirmed resorption of the bone underneath the implant (**C**). The surgical plan called for the removal of the silicone implant and immediate placement of a custom-made PEEK chin implant to augment the anterior lower third and create a squarer, more masculine appearance (**D**).

**Figure 4:**
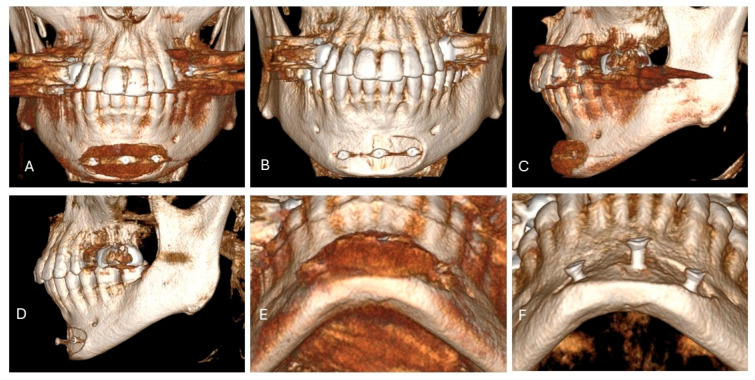
A 31-year-old Caucasian man presented for consultation because he was dissatisfied with a previously placed stock silicone chin implant stabilized with titanium screws (**A**–**F**). Although the patient had undergone comprehensive dental and orthodontic treatment, he had a “weak” mandible and jawline from untreated Class II malocclusion. CT showed that, despite bony fixation with titanium screws, the implant had caused mild bone resorption at the implant interface (**B**,**D**,**E**). The treatment plan called for removal of the silicone implant and screws, followed by immediate masculinizing genioplasty and placement of custom mandibular angle implants according to our protocol for lower-third masculinization.

**Figure 5:**
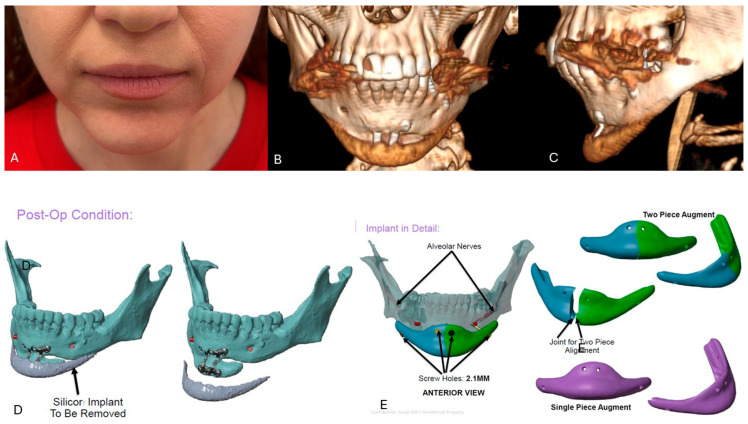

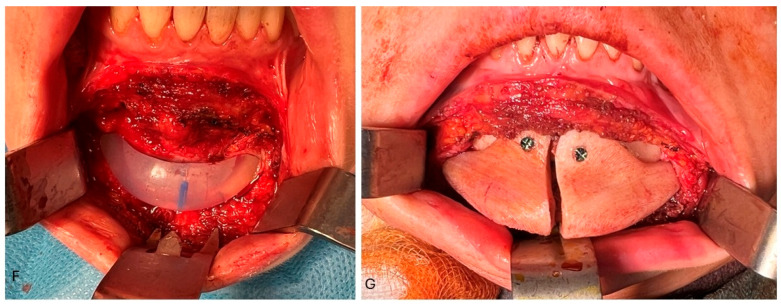
Clinical photographs of a 35-year-old Caucasian woman who presented for consultation because of asymmetry and imbalance of the lower third after prior surgery elsewhere (**A**). She had a severe Class II malocclusion previously managed with camouflage orthodontics, followed by sliding genioplasty and implant placement one year later (**B**,**C**). At the time of consultation, she complained of asymmetry and hollowness at the lateral aspects of the chin resulting from anterior augmentation confined to the central area (**A**). The treatment plan called for removal of the silicone implant (**D**,**F**) and augmentation with a custom two-piece chin implant stabilized with titanium screws (**E**–**G**).

Among 98 patients who attended for primary or secondary procedures with the use of silicone facial implants, 25 of them (11 males—44%, 14 females—56%) exhibited complications and were enrolled into the study (Table 1). Of the analyzed patients, 21 had implants placed elsewhere and were referred for consultation or revision surgery. Indications for chin implant placement were purely esthetic (*n* = 10), camouflage of Class II dental deformity (*n* = 10), or masculinization of the lower third (*n* = 3). The most commonly encountered complications that led to revision surgery were displacement (*n* = 3) (12%), bone resorption (*n* = 9) (36%) or both (*n* = 3) (12%), and patient dissatisfaction (*n* = 7) (28%). Displacement was diagnosed in cases where no rigid implant fixation was applied. Bone resorption ranging from mild to severe was seen in both fixed and non-fixed implants (Figure 1, Figure 2, Figure 3 and Figure 4). Dissatisfaction was met in the group of patients who underwent chin augmentation with silicone implants due to Class II dentofacial deformity or if they wanted to masculinize their jawline. All patients were qualified for revision surgery: silicone implant removal with subsequent sliding genioplasty (*n* = 7) (28%), orthognathic surgery (*n* = 4) (16%), custom-made chin implant placement (*n* = 7) (28%), and reposition and fixation (*n* = 1) (4%) (Figure 3 and Figure 5).

## 4. Discussion

Silicone implants have been used in facial surgery since the 1960s and were considered to be one of the safest alloplastic materials, easy to use, and with good results. However, due to increasing reports of complications regarding their longitudinal interference with host tissues, some concerns have been raised regarding their “universal” applications, especially in craniomaxillofacial and esthetic surgery. One of the most commonly discussed issues regarding implants is their displacement due to inadequate fixation, extrusion, bone resorption, and/or spur-like neo-ossification based on subperiosteal bone induction by periosteal distraction [1,7,8]. Some studies also describe alteration of facial muscles and lip dysfunction [9]. This was not observed in the studied cohort.

Implant displacement is associated with a lack of proper stabilization beneath the tissues. In the past, silicone chin implants were routinely placed via submental or intraoral incision and sutured with surgical sutures or wires. Insufficiently stabilized implants may displace, leading to facial asymmetry and/or bone alterations due to micromovements, which was also seen in six presented cases who required revision surgery [3,10]. A chin implant should be placed at the lower border of the mandible and fixed with at least one screw in order to prevent movement. In other cases, it may rotate or be displaced inferiorly or superiorly, leading to resorption of the cortical bone covering the root apices and/or result in inadequate projection, leading to patient dissatisfaction, which was also reported in this manuscript. When not stabilized, it may also result in ectopic bone formation, which consequently also leads to asymmetry and patient dissatisfaction. Also, implants should be placed passively, without any pressure on the bone, as it may induce bone resorption, as was proven by Pearson and Sherris (1999) [11]. Also, the size of the implant should be as small as needed, as, along with implant size, the risk of bone resorption is increasing according to the study of Wellisz et al. (1995). Also, in the study of Yeung & Wong (2022), it was shown that mandibular bone resorption associated with chin implants highlighted the greatest resorption rates in softer bone types (like Type 4) compared to denser types (Type 1–3) [12]. Their results indicated a correlation between bone density and resorption outcomes with use of silicone chin implants [12].

When a too-big implant is used, or a too-small dissection is performed, soft tissue coverage may be insufficient, and implant extrusion may occur. Such scenarios require implant removal with subsequent revision surgery at least a few weeks thereafter. Therefore, silicone chin implants should not be routinely used as camouflage for class II dentofacial deformity, especially in severe cases. This is because they may be insufficient for camouflage, and the effect obtained is unnatural. Also, they may cause bone resorption and the necessity of implant removal with subsequent orthognathic surgery, which is needed in the first place, as seen in the presented cohort. In one presented case, a silicone chin implant was placed onto a bone segment after previously performed genioplasty, both done elsewhere. Such an approach resulted in an unnatural “witch’s chin” deformity. The patient refused orthognathic surgery and thus, a custom extended chin implant was placed in order to restore facial balance (Figure 5).

Bone resorption induced by foreign body response is one of the most discussed issues regarding silicone facial implants. Robinson was the first to describe the process of resorption under silicone and acrylic chin implants, attributed to foreign body giant cell reaction between the implant and the bone or to pressure from the mentalis muscle against the implant (Robinson, 1972) [13]. The study also revealed that resorption showed up to 3–5 mm at an average of 30–48 months postoperatively, which corresponds to 0.1 mm per month.

Significant resorption poses not only an obvious problem associated with the creation of a bony defect and potential damage to underlying structures like tooth roots, but it also leads to loss of chin projection, which was also seen in the presented cohort [9]. Hence, a debate about implant positioning and its stabilization has gained attention throughout the last 60 years. Historically, silicone implants have been stabilized by percutaneous temporary sutures as described by von Szalay or Peled, but they did not provide sufficient implant placement, which is still considered one of the factors inducing bone resorption (Peled & Szalay, 1994; Szalay, 1992) [14,15]. Nowadays, subperiosteal implant placement with rigid stabilization with the use of titanium screws is considered a gold standard, yet it still poses some limitations for the use of silicone in the craniofacial area and is associated with the distinct anatomy and physiology of the orofacial region. The mechanism of bone resorption was evaluated by Pearson and Sherris (1999) [11]. An animal model was used to indicate whether the sub- or supraperiosteal position of the implant promotes osteoclastogenesis. The authors did not observe a significant difference in bone resorption between the sub- or supraperiosteal position of the implants and concluded that some level of resorption is inevitable when silicone implants are applied. The authors concluded that the mechanism of bone resorption under implants that were placed supraperiosteally results from pressure-induced necrosis of the periosteum under the implant. In the case of implants placed directly onto the bone, resorption was a result of periosteal disruption [11]. From a clinical point of view, subperiosteal placement is more precise, gives better implant alignment with bony curvature, and better mimics natural tissue projection. Also, subperiosteal placed implants are less palpable and tend to migrate in a lesser range than the supraperiosteal position. Also, subperiosteal placement helps to prevent implant exposure.

The placement of chin implants supraperiosteally or without fixation allows for micromovement due to activation of the mentalis. This may lead to erosion into the body of the mandible and/or chronic infection with extrusion [9]. Bone resorption was also a major causative factor for revision surgery within the presented study, based on secondary sliding genioplasty, adapting 3D custom-made implants or orthognathic surgery. Resorption was diagnosed in both rigid-fixed and non-fixed implants. However, there were no cases with implant migration when rigid fixation was applied. There was one dramatic case, where a superiorly displaced implant induced bone resorption up to the periapical spaces of the teeth. The patient was referred for implant removal with immediate bone augmentation and subsequent orthognathic surgery. This case illustrates clearly when cosmetic procedures such as camouflage orthodontic treatment alongside “minor” esthetic surgery are an inappropriate way of treatment. Overall, non-fixed silicone chin implants are associated with an 18.25% bone loss, versus only 1% in fixated implants, according to the current literature [7,16,17]. Hence, in every operated case performed by the Author (primary or revision cases) rigid fixation was applied. According to Madorsky & Meltzer (2025) [7], subperiosteal placement may, however, induce more complex sequelae of processes that lead to implant “entrapment” under the periosteum, which is induced to promote spur-like neo-ossification based on subperiosteal distraction, which gives a new shade on the conceptualizations based on silicone facial implants. Bone formation between the elevated periosteum and native bone is believed to be induced by distraction osteogenesis mechanisms. This may lead to de novo bone formation above the implant or underneath. New bone forming on top of the implant may induce its compression on the opposing site and bone resorption, leading to implant displacement and asymmetry, which was also seen in one case presented within this study [7].

Early infections and the necessity of implant removal are rarely seen in silicone chin implants due to the smooth surface being unfavorable for bacterial biofilm formation when compared to other materials. The overall infection rate of silicone chin implants is estimated at 0.7%. However, there were unusual infections of the implants described, secondary to dental procedures. In the study by Danieletto-Zanna et al. (2022) [1], an infected silicone chin implant had to be removed due to the nonideal placement of dental implants, which developed a bacterial infection [1]. In another study, an implant had to be removed due to an odontogenic infection spreading into the peri-implant space (Hoffman, 1981) [18]. In the presented study, there was only one case of implant infection secondary to esthetic procedures performed in the near proximity of the already healed implant, which, in consequence, required removal despite attempts being made to preserve it. These clinical situations clearly show the role of microflora of the oral cavity and risk factors associated with implant contamination from odontogenic or sinus infections (active or developed after implant placement) [19,20].

Among the investigated cohort, there was only one case of implant infection, not related to the surgery itself. It was caused by additional procedures with the use of hyaluronic acid injection at the top of the implant, performed elsewhere two years after implant placement, which caused bacterial biofilm inoculation. Despite efforts being made based on implant debridement and general antibiotic therapy, the implant had to be removed with no procedures performed thereafter. It is evident that any additional procedures based on injectables in the near proximity of the implant may unintentionally be a causative factor if peri-implant infection, and thus, its removal was also seen in the presented cohort [21].

Patient dissatisfaction was also another cause for revision surgery. This was common in patients with severe Class II dental malocclusion or those who desired masculinization of the lower third. In these cases, either custom-made PEEK implants, genioplasty, or orthognathic surgery was applied. This clearly shows that, along with the development of surgical techniques and 3D modeling, silicone facial implants are gradually being considered less universally applied than before, especially in cases of dentofacial deformities or gender-affirming procedures.

This retrospective study exhibits some limitations. The cohort was heterogeneous; some patients were operated on by different surgeons elsewhere and came for a second opinion and thus, revision surgery. The minimum follow-up for all operated patients (primary or secondary) was six months. However, some patients had implants placed years before the consultation, which resulted in revision surgeries described within this study. In some cases, resorption was asymptomatic and was diagnosed at the time of consultation, making it impossible to assess exactly at which point it began. Additionally, there is currently no information available about the type of bone quality and its tendency for resorption after contact with silicone implants. The current literature does not provide sufficient information on how the age of the patients impacts the tendency for bone resorption after direct contact with silicone implants.

## 5. Conclusions

Silicone facial implants still serve as a versatile tool in esthetic facial surgery. However, due to their tendency to induce bone resorption, their use should be judiciously evaluated. They are of great use in minor tissue augmentation in aging faces, and if used, they should be placed in the subperiosteal plane with rigid fixation in order to prevent displacement and bone resorption. These implants should not be routinely used in the camouflage of dentofacial deformities, where sliding genioplasty or orthognathic surgery should be performed in the first place. As stock implants, they are also not found to be a reliable way in gender affirming surgery. In more complex clinical cases or when larger implants are required, 3D solid implants that provide precise alignment within the tissues and better esthetic outcome should be applied.

## Figures and Tables

**Table 1:** Characteristics of the patients enrolled in the study, evaluating indications of the primary surgery, complications, fixation used, and secondary procedures that were performed. Gender: M—male, F—female.

Patient	Gender	Age (Years)	Indications	Primary	Stabilization with Screws	Complications	Secondary	Plan
1	M	28	Masculinization	no	no	Bone resorption	yes	Removal and placement of PEEK custom chin implant
2	F	38	Esthetic	no	no	Displacement and resorption	yes	Removal
3	M	25	Esthetic	no	yes	Bone resorption	yes	Removal
4	M	31	Esthetic	no	no	Dissatisfaction	yes	Removal and placement of PEEK custom chin implant
7	F	29	Class II dentofacial deformity	no	no	Bone resorption	yes	Orthognathic surgery, augmentation
8	M	32	Class II dentofacial deformity	yes	yes	Bone resorption	yes	Orthognathic surgery, bone augmentation
9	M	35	Class II dentofacial deformity	no	yes	Bone resorption	yes	Orthognathic surgery, PEEK angle implants
10	F	29	Class II dentofacial deformity	no	no	Dissatisfaction	yes	Removal and placement of PEEK custom chin implant
11	F	45	Esthetic	no	no	Displacement	yes	Repositioning and stabilization with screws
12	F	41	Esthetic	yes	yes	Infection	yes	Removal
13	M	27	Class II dentofacial deformity	no	yes	Bone resorption	yes	Removal, sliding genioplasty
14	F	45	Esthetic	no	no	Displacement	yes	Removal, sliding genioplasty
15	M	30	Class II dentofacial deformity	no	no	Bone resorption	yes	Removal and placement of PEEK custom chin implant
16	F	19	Class II dentofacial deformity	no	no	Bone resorption	yes	Removal, sliding genioplasty
17	F	51	Esthetic	no	no	Displacement and resorption	yes	Removal, sliding genioplasty
18	M	20	Masculinization	no	yes	Bone resorption	yes	Removal and placement of PEEK custom chin implant
19	F	36	Esthetic	yes	yes	Dissatisfaction	yes	Removal and placement of PEEK custom chin implant
20	F	32	Esthetic	no	no	Displacement	yes	Removal, sliding genioplasty
21	M	25	Class II dentofacial deformity	no	no	Dissatisfaction	yes	Orthognathic surgery
22	F	27	Class II dentofacial deformity	no	no	Dissatisfaction	yes	Removal, sliding genioplasty
23	F	36	Esthetic	no	no	Displacement, resorption	yes	Removal, custom PEEK chin implant
24	M	37	Masculinization	no	yes	Dissatisfaction	yes	Removal, custom PEEK chin implant
25	M	40	Class II dentofacial deformity	no	no	Dissatisfaction	yes	Removal, sliding genioplasty

## Data Availability

There is no other data than presented within this manuscript.
